# Influence of Different Bracket Adhesive Systems on Enamel Demineralization—An In Vitro Study

**DOI:** 10.3390/jcm12134494

**Published:** 2023-07-05

**Authors:** Christoph-Ludwig Hennig, Simon Löhnert, Ann Nitzsche, Sandor Nietzsche, Frank Steiniger, Justus Marquetand, Konrad Tolksdorf, André Guellmar, Bernd Sigusch, Collin Jacobs

**Affiliations:** 1 Department of Orthodontics, Center of Dental Medicine, University Hospital Jena, An der Alten Post 4, 07743 Jena, Germany; 2 Electron Microscopy Center, University Hospital Jena, Ziegelmühlenweg 1, 07743 Jena, Germany; 3 Department of Epileptology, Hertie-Institute for Clinical Brain Research, University of Tübingen, 72074 Tübingen, Germany; 4 Department of Neural Dynamics and Magnetoencephalography, Hertie-Institute for Clinical Brain Research, University of Tübingen, 72074 Tübingen, Germany; 5 MEG-Center, University of Tübingen, 72074 Tübingen, Germany; 6 Department of Oral and Maxillofacial Surgery/Plastic Surgery, University Hospital Jena, Am Klinikum 1, 07747 Jena, Germany; 7 Department of Conservative Dentistry and Periodontology, Center of Dental Medicine, University Hospital Jena, An der Alten Post 4, 07743 Jena, Germany

**Keywords:** orthodontic bracket adhesive, white spot lesion, tooth demineralization, bacterial adhesion, biofilm formation, flash-free brackets

## Abstract

Background: enamel demineralization is a common side effect of orthodontic therapy with fixed braces. The aim of the present in vitro study was to compare a conventional adhesive system and a modern adhesive system (APC Flash-Free [FF] technology) with regard to the demineralization of enamel by *Streptococcus sobrinus* (*S. sobrinus*). Methods: this in vitro study included premolar teeth and compared APC FF adhesive brackets (Group A, *n* = 15) with conventional adhesive brackets (Group B, *n* = 15) from the same company. Specimens were incubated with a positive control group (PCG, *n* = 5) and a negative control group (NCG, *n* = 5) in an *S. sobrinus* suspension for three weeks. To evaluate the grade of enamel demineralization, the samples were analyzed using a polarizing microscope. Results: the test specimens of group B with conventionally bonded bracket adhesive showed significantly greater (+10.8 μm) demineralization with regard to the penetration depth of the demineralization than the PCG (*p* = 0.012). Thus, there was a difference from group A with the new bracket adhesive of the FF brackets (+7.29 μm). Significantly, demineralization was more pronounced cervically than coronally in both groups, and it occurred cervically more frequently than grade 3 demineralization (*p* = 0.001). Conclusions: it seems plausible that new orthodontic bracket adhesives and the modern FF adhesive system positively contribute to the reduction in enamel demineralization during orthodontic treatment.

## 1. Introduction

Orthodontic treatment often involves the use of fixed treatment appliances. These are applied to the surface of teeth using certain adhesive systems. The adhesion between the tooth tissue and the dental material depends on various factors. The surface and the adhesive are decisive for the bonding between the tooth and the bracket. The correct preparation of the surfaces is ensured by roughening with phosphoric acid and bonding systems [1]. However, the bond between the teeth and the orthodontic appliance has the disadvantage that the teeth are more difficult to clean. In addition, the fixed appliance significantly increases the retention of food debris [2]. Oral hygiene is more difficult, which leads to the increased formation of bacterial plaque [3,4], and, during orthodontic treatment, the side effect of enamel demineralization can occur. Initially recognizable as so-called “white spot lesions”, shimmering white decalcifications, which are initially superficially limited, but which can develop into profound carious lesions if plaque persists, make restorative measures necessary [5]. White spots considerably impair one’s esthetic appearance and are also irreversible. The formation time of these white spot lesions usually lasts only a few weeks [6]. To prevent white spot lesions, dental hygiene measures are most important, supported by fluoridation or sealing of the enamel surface. There are also some sealants on the market that can effectively protect the enamel from demineralization [7]. However, studies have shown that chemical and mechanical stress can weaken these sealants. Interruptions in the sealant layer can lead to demineralization under the sealants [8].

In addition to the preventive methods mentioned above, there is another component that influences bacterial adhesion—the spatial nature of the adhesive system [9]. Ideally, the transition from enamel to bracket should be as smooth as possible, so that bacterial adhesion is prevented as much as possible by the lack of retention spaces and patient hygiene is simplified. A new adhesive technology (APC Flash-Free [FF] Adhesive Coated Appliance System, 3M Unitek, Monrovia, CA, USA) has eliminated the need for excess adhesive material removal during bracket bonding compared to the standard bonding technique. The adhesive layer is incorporated in a fiber matrix on the bracket base precoated by the manufacturer. The aim in the present study was to determine the extent to which the chamfered marginal seam created by the FF adhesive system reduces bacterial adhesion and thus prevents demineralization of the enamel. The study was carried out in vitro and compared the FF adhesive system with brackets that were conventionally adhesively bonded under basic conditions, which were as standardized and reproducible as possible. The demineralization was triggered by inoculation with *Streptococcus sobrinus*, the main bacteria that colonizes oral surfaces in the occurrence of cariogenesis [10,11].

## 2. Materials and Methods

### 2.1. Tooth Selection and Preparation

Extracted teeth were collected from dental and oral surgery practices and subsequently sorted for suitability for the study. The teeth were manually cleaned with water after extraction, disinfected in alcohol for 2 min, and stored in 0.9% sodium chloride solution. As intact enamel is essential for the experimental procedure, any teeth with caries, fluorosis, enamel cracks, white spot lesions, anatomical anomalies, enamel spalling due to extraction, and iatrogenic restorations were excluded. The teeth were examined under a JenaPol polarizing microscope (Carl Zeiss, Jena, Germany) by a dentist. Only teeth with intact and caries-free enamel surfaces and free of enamel structural defects were selected. The patients gave their consent to release the extracted teeth for the study. The consent of the ethics committee of the medical faculty at the University of Jena was also obtained (no. 2021-2486_1).

The teeth were manually cut along the longitudinal fissure from the mesial direction to the distal direction with a diamond-coated disc (355 ZRO-Diamantscheibe, 22.0 mm, accurata GmbH, Thurmansbang, Germany) with the dental mikromotor AM-25 A RM (WH Dentalwerk, Bürmoos, Austria) under water cooling at 12.000 rpm. In a second cut, the roots of the teeth were separated horizontally in the area just below the enamel–cement interface and embedded in epoxy resin (EpofixResin, Struers GmbH, Willich, Germany).

### 2.2. Production of the Test Specimens

To avoid contamination with foreign germs, the test specimens were autoclaved for 20 min at 121 °C in the autoclave Vacuklav 40 B+ Evolution (MELAG Medizintechnik GmbH & Co. KG, Berlin, Germany). The following steps were performed on a sterile workbench with a suction unit. The teeth were placed on the work surface under the suction unit to dry. After 1 h, the test specimens were sufficiently dry to start bonding the brackets. For both bracket adhesive systems, the enamel was prepared with Transbond TM Plus Self Etching Primer (3M Unitek, Monrovia, CA, USA). The mixing process was carried out according to the manufacturer’s instructions. Transbond TM Plus Self Etching Primer was applied to the area of the tooth intended for the bracket and massaged in for 5 s on the tooth surface. The bonding was then lightly blown for approximately 2 s and then lightly polymerized for 20 s. An Acteon Mini LED polymerization lamp (Acteon Germany GmbH, Düsseldorf, Germany) was used for this purpose.

For group B (*n* = 15), Clarity Advanced ceramic brackets (3M Unitek, Monrovia, CA, USA) were removed from the packaging, and the bracket bases were coated with Transbond XT Ligth Cure Adhesive Paste (3M Unitek, Monrovia, CA, USA), and the brackets were positioned on the teeth with moderate pressure using bracket tweezers. The resulting excess was removed using a Heideman spatula (Aesculap Dental Instruments, B. Braun SE, Melsungen, Germany), and then the brackets were light-polymerized from the mesial and distal sides for 20 s. For group A (*n* = 15), the FF brackets (3M™ Victory Series™ Low Profile Bracket System (3M Unitek, Monrovia, CA, USA) were removed from the opaque packaging, positioned on the pretreated teeth using bracket tweezers, and fixed with moderate pressure. Subsequently, light polymerization was performed for 20 s from each side. In addition, a positive control group (PCG) (*n* = 5) and a negative control group (NCG) (*n* = 5) were created. The PCG was included in the bacterial suspension without gluing a bracket. The NCG was only placed in the culture medium. After application of the brackets and before inoculation with *S. sobrinus*, UV sterilization was performed for 30 min on the sterile workbench (Thermo Fisher Scientific, Waltham, MA, USA) to prevent contamination with foreign germs. Thus, a total of forty teeth were produced as test specimens—fifteen for group A, fifteen for group B, and five each for the PCG and NCG.

### 2.3. Bacterial Culture

*S. sobrinus* (strain no: DSM 20742 dated 05/19) (Leibniz Institute, DSMZ-Deutsche Sammlung von Microorganisms and Cell Cultures GmbH, Brauschweig, Germany) was thawed, and a 48 h culture was established on a blood agar plate (Carl Roth GmbH, Karlsruhe, Germany). One to two colonies were collected and suspended in 10 mL Schaedler bouillon (Carl Roth GmbH, Karlsruhe, Germany) and then cultured again for 24 h. The culture was then centrifuged at 3500 u/min for 5 min, and the supernatant was discarded. The sediment was mixed with 1 mL Schaedler bouillon (Carl Roth GmbH, Karlsruhe, Germany) and further diluted with Schaedler bouillon (Carl Roth GmbH, Karlsruhe, Germany) to prepare an inoculum with an optical density of 0.01 from this bacterial batch. 

Next, all samples of groups A and B and the PCG were inoculated with 1 mL of the bacterial inoculum and 2 mL tryptic soy broth. The NCG was filled with 3 mL pure Schaedler bouillon (Carl Roth GmbH Karlsruhe, Germany) per well. A check of the pH of the solution showed a value of 7 at the beginning.

The well plates were stored in an anaerobic box (MKIII Fa., Meintrup DWS) at 37 °C, 80% N_2_, 10% CO_2_, and 10% H_2_. The medium was changed once a week, removing 2 mL from each well and adding 2.5 mL Schaedler bouillon (Carl Roth GmbH, Karlsruhe, Germany) to compensate for any evaporated medium. All test specimens were incubated in the anaerobic box for 3 weeks.

To control the bacterial growth of *S. sobrinus*, a determination of the colony-forming units was made at each medium change. The determination was made from any well, 1× per group. For this purpose, 10 µL bacterial preparation plus 990 µL Schaedler bouillon (Carl Roth GmbH, Karlsruhe, Germany) were diluted (i.e., 1:100). Of this, 20 µL were spatted out on Schaedler bouillon (Carl Roth GmbH, Karlsruhe, Germany) or agar plates (Carl Roth GmbH, Karlsruhe, Germany). For the negative control, 20 µm were spatted directly onto Skinner agar plates. After 3 d, the colonies were counted and calculated as follows: number of colonies × 50 (factor converted 20 µL to 1 mL) × 100 (dilution 1:100).

### 2.4. Methodology of Evaluation

After 3 weeks, the test specimens were removed from the solution. The slices were rinsed with NaCl. Bacteria were fixed in 4% paraformaldehyde (PFA) (Thermo Fisher Scientific, Waltham, MA, USA) for 48 h at approximately 4 °C. A repeat pH control of the bacterial suspension revealed a pH value of 5.0 for six randomly selected wells of group A and group B. The pH value of the negative controls remained stable at 7.0. The pH control was performed with buffers from Thermo Fisher (Thermo Fisher Scientific, Waltham, MA, USA). After 48 h, the PFA was rinsed from the specimens with NaCl. 

The group A and B brackets were carefully detached from the teeth using direct bond and bracket removal forceps (Carl Martin GmbH, Solingen, Germany). The bonding was left in place. Special attention was paid to keeping the enamel surface intact in order not to damage the surface under observation. After careful removal, all specimens were stored in 20 mL of distilled water at approximately 4 °C. The epoxy resin (EpofixResin, Struers GmbH) in which the teeth were initially embedded was removed. This was performed manually using two flat forceps. 

For analysis, by means of a polarization microscope haircut, a thickness of 80–100 μm was required. This was achieved with two saw microtomes of the Leica type SP1600 (Leica Mikrosysteme Vertrieb GmbH, Bensheim, Germany). Due to the surface area, approximately 16 sections on a test specimen can be evaluated in the polarization microscope. With a total of 40 test specimens, this would theoretically be 640 measuring points. 

Cross-sectional images were obtained showing the teeth tilted 90° in cross-section from the cusp tip to beyond the enamel–cement interface. Microscopy was performed with a JenaPol polarizing microscope (Carl Zeiss, Jena) at 30× magnification. The sections were photographed using an AxioCam microscope camera (Carl Zeiss, Oberkochen, Germany). The sections were evaluated using Axiovision software, version 4.8.2.0 (Carl Zeiss MicroImaging GmbH, Oberkochen, Germany). The choice of magnification allowed exact scaling on the recorded image and, thus, a micrometer-precise measurement of the demineralization depth.

For the evaluation, the field to be considered was set at 1000 μm from the beginning of the transition of the bracket adhesive to the enamel in the direction of the periphery. Demineralization was measured from the deepest point of penetration to the enamel surface. Because demineralization was often similar in depth, but varied in severity, each measured case of demineralization was assigned a severity level, and, for this purpose, a classification into demineralization grades was developed for this study:

Grade 0 = No demineralization

Grade 1 = Incipient demineralization

Grade 2 = Demineralization with strongly visible lesion body

Grade 3 = Demineralization with clearly visible lesion body and broken surface

**Figure 1:**
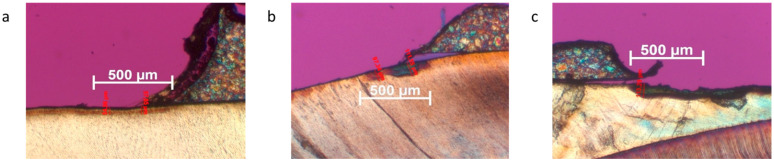
The severity of demineralization: (**a**) Grade 1 = incipient demineralization; (**b**) Grade 2 = demineralization with strongly visible lesion body; and (**c**) Grade 3 = demineralization with clearly visible lesion body and broken surface.

Figure 1 shows the severity of demineralization (Figure 1).

### 2.5. Statistical Analysis

Descriptive statistics were performed for both groups, and median, as well as interquartile ranges, were calculated. Data distribution was assessed with the Kolmogorov-Smirnov normality test. The Mann–Whitney U test for pairwise comparisons was used for non-parametric testing, as data did not pass the normality test. A two-sided *p*-value < 0.05 was considered significant. Statistical analyses were conducted in SPSS (IBM, Armonk, NY, USA). 

## 3. Results

The procedure is generally feasible and suitable for the detection of demineralization in the bracket environment. Incipient demineralization was particularly evident in the area of the transition from the bracket adhesive to the enamel. All groups (A, B, and PCG) that were placed in a bacterial suspension showed slight demineralization. The NCG showed no demineralization. There is no evidence to suggest that the bacterial medium used in this experiment has a demineralizing effect on the surface of human enamel.

Of the theoretical 640 measured values to be collected, 100 measuring points could not be evaluated. This was mainly due to melt fractures that occurred during the production of the fine sections in the saw microtome. A total of 540 measured values for the depth of demineralization were included in the statistical evaluation. Regarding the severity of demineralization, differences were found between the bracket groups and the PCG.

**Figure 2:**
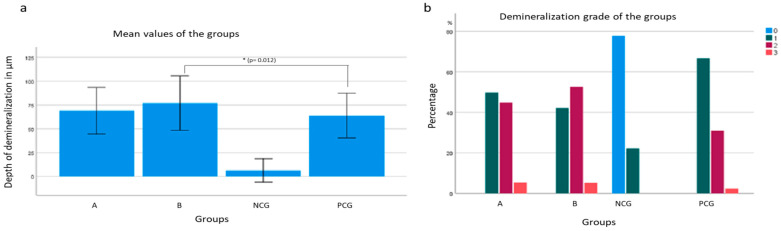
Enamel demineralization from group A, group B, PCG (positive control group) and NCG (negative control group): (**a**) mean values of the depth of demineralization of the groups with significance from group B to PCG (*p* = 0.012); (**b**) percentage distribution of the degrees of demineralization in the groups. * *p* < 0.05.

In group A, where the test specimens were bonded with FF brackets, a mean penetration depth of 68.84 μm was determined from 242 measurement points. Of these measurement points, 49.8% were grade 1, 44.8% were grade 2, and 5.4% were grade 3. Thus, the penetration depth increased by an average of 3.54 μm compared to the PCG. No statistical significance (*p* = 0.402) was found for this mean difference. Compared to the PCG, grade 1 demineralization decreased by 16.8%, grade 2 demineralization increased by 13.8%, and grade 3 demineralization increased by 3%. The conventionally bonded brackets in group B had a mean penetration depth of 76.14 μm at 211 measurement sites. This value increased by 10.83 μm compared to the PCG. Significance was found in this mean difference at *p* = 0.012 (Figure 2). The proportions of demineralization were as follows: 42.2% grade 1, 52.6% grade 2, and 5.2% grade 3. This means, compared to the PCG, there was 24.5% less grade 1 demineralization, 21.6% more grade 2 demineralization, and 2.4% more grade 3 demineralization.

The demineralization depths of both bracket groups (A and B) increased on average compared to the PCG (not bonded with brackets) (Figure 2). In this study, only the difference of group B compared to the PCG was shown to be significant (*p* = 0.012). Both bracket groups A and B had a higher chance of grade 2 demineralization than the PCG (Figure 2). The odds ratio for group A was 2.438, and, for group B, it was 3.332. The result was significant for both groups.

For grade 3 demineralization, a lower probability was found for the bracket groups than for the PCG. This result is not significant with *p* = 0.808 for group A and *p* = 0.850 for group B and is due to the low number of readings in the PCG. Demineralization-free sections could only be microscoped in the NCG (Figure 2). Only the depth of demineralization and the severity of demineralization differed. The test specimens of group A, which were bonded with FF brackets, had a lower penetration depth with an average demineralization depth of 68.84 μm compared to the test specimens of group B, which had an average demineralization depth of 76.14 μm. The difference of 7.298 μm represents a difference between the two groups (Figure 2). The probability of grade 2 demineralization was 0.736 times lower in group A compared to group B. The result was not statistically significant at *p* = 0.151. There was a minimally higher probability of grade 3 demineralization in group B. The chance of grade 3 demineralization was 0.935 times lower in group A than in group B. Again, the result was not significant at *p* = 0.899 (Figure 2).

**Figure 3:**
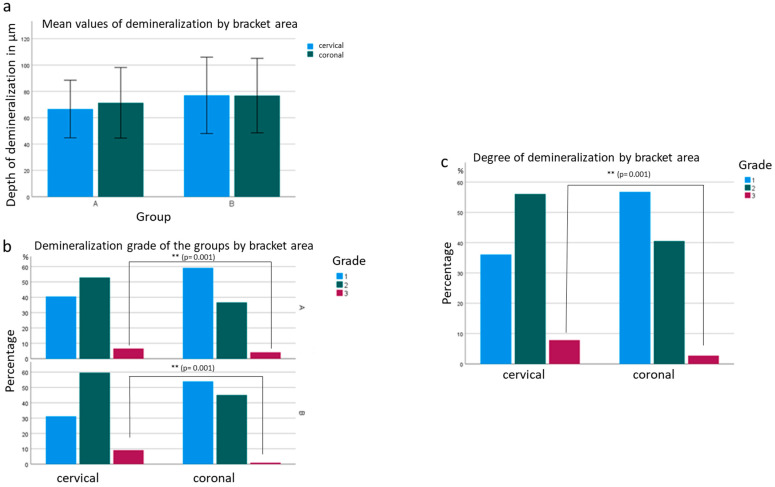
Enamel demineralization by bracket area localization (cervical, coronal): (**a**) mean values of depth of demineralization by bracket area between groups; (**b**) degree of demineralization of groups by bracket area (*p* = 0.001); (**c**) percentage distribution of degrees of demineralization by bracket area from both groups (*p* = 0.001). ** *p* < 0.01.

Furthermore, it was noticed that the depth of demineralization at the different locations of the bracket (cervical, coronal) was different. Regarding the penetration depth, the demineralization at the coronal junction of adhesive and enamel was, on average, 2.091 µm deeper than at the cervical junction (Figure 3). There was a statistical significance between the bracket systems as to whether demineralization occurred more frequently at the coronal or cervical transition of the adhesive to the tooth structure. In group A, the depth of demineralization was lower, but more demineralization occurred in the coronal transition of the adhesive to the enamel. In group B, there was no difference between the coronal and cervical areas. Regardless of group, cervical enamel demineralization increased by 2.657 times compared to coronal enamel demineralization. For grade 3 demineralization, the probability increased by 10.028 times. Both results are significant at *p* = 0.001 (Figure 3).

## 4. Discussion

The insertion of a multiband appliance increases the occurrence of demineralization in relation to the penetration depth and severity of demineralization. There are different strategies of modern adhesive systems to reduce the demineralization of the enamel. These adhesive systems are often combined with sealers for smooth surfaces. It is, therefore, obvious that smooth surfaces and a homogeneous adhesive layer have a positive effect on the structural surface and reduce the demineralization of the enamel. One possible approach that could reduce the demineralization which occurs on tooth surfaces, is to use brackets with precoated adhesive, as this provides the optimal amount of adhesive on the bracket base, results in less excess adhesive, and therefore less bacterial accumulation. This has already been shown in a study, where less excess adhesive in the area around the bracket reduces the adhesion surface, and, therefore, fewer bacteria accumulate in the bracket periphery [12]. In addition to the FF brackets, the development of adhesive systems has resulted in so-called APC PLUS brackets. These are pre-coated with an adhesive that creates a flash when placed. This appears pink in color before light polymerization, making it easier for the orthodontist to remove the flash. After light polymerization, the adhesive changes color from pink to clear. In 2007, Armstrong et al. demonstrated that staining the adhesive did not result in reduced residual flash compared to conventional adhesives.

Therefore, practitioners had to be vigilant in removing any excess to prevent plaque retention, gingival irritation, and white spots [13,14,15]. FF adhesives use a low-viscosity adhesive that is applied to a non-woven material made of polypropylene fibers. This special fabric is cut exactly to the size of the bracket. The fabric is easily compressible and can thus adapt to the individual anatomical tooth surface without expanding laterally to avoid the formation of excess [16]. Furthermore, the transition from bracket to tooth is morphologically shaped in the form of a chamfer, thus enabling better hygienity of the inserted appliance. The versatile improvements of the FF adhesive system claimed by the manufacturer 3M have already been investigated in several studies. Jung et al. compared the morphology of the APC PLUS flash with that of FF brackets and found no significant differences. In terms of adhesive layer thickness, the adhesive of the FF brackets was found to be thicker, but also more homogeneous, than the adhesive of the APC PLUS brackets [17]. Shortened bonding times were demonstrated in comparison with conventional adhesives. Both per tooth (37.3%) and per quadrant (32.9%), the bonding time was significantly reduced. Regarding bracket survival, no significant differences were observed over the one-year period [18]. Lee and Kanavakis reconfirmed the shortened workload and demonstrated increased bonding strength against shear forces [19]. According to Grünheid and Larson, the FF adhesive also saves time after orthodontic treatment. Because less adhesive remained after bracket removal, the amount of work required for adhesive removal was reduced by 20% [20]. Foersch et al. were able to demonstrate a reduction in the time required to apply the FF brackets compared to the APC Plus brackets. Furthermore, they observed an extension of the adhesive beyond the bracket base of 0.16–0.08 mm, but this excess appeared smooth and sufficient with regard to the transition of the enamel, possibly allowing reduced bacterial adhesion [21].

Our study relates to the demineralization of enamel, which is caused by bacterial biofilm accumulation on the materials, particularly on the bracket adhesive. The nature of the material, as well as the localization and size of the material, plays a decisive role in biofilm deposition. As early as 1979, electron microscopic investigations of biofilms formed around brackets revealed that biofilms preferentially form on roughened surfaces at the transition from enamel to bracket adhesive. Particularly mature plaque was found gingival to the bracket [9]. In addition to a rough surface, the formation of a gap space at the composite–enamel interface is an additional risk factor for bacterial plaque formation [22]. Regarding these observations, it was found, in 2019, that the microbial composition of the biofilm around FF brackets was altered compared to APC pre-coated brackets. The biofilm around FF brackets showed a reduced number of pathogenic bacteria, as well as a reduced total bacterial count. That the use of FF brackets affects demineralization in the bracket environment could not be confirmed [23].

The present study shows that, with regard to the amount of demineralization that occurred, there was measurable demineralization in groups A and B and the PCG in every evaluable section. The choice of different bracket adhesive systems can, therefore, only influence the formation of demineralization and not prevent it. Therefore, it is essential to maintain sufficient oral hygiene, regardless of the brackets or adhesives used. The demineralization in the PCG was intermediate with a penetration depth of 65.31 μm. Demineralization was found in all 42 sections; 66.7% of the demineralization was grade 1.31% was grade 2, and 2.4% was grade 3. This was a significant increase compared to the NCG, which proves that the measured demineralization was caused by metabolic products of *S. sobrinus* during the experimental procedure. According to the preliminary tests, the measured values were in the targeted interval between 50 μm and 100 μm; this is in agreement with the research results of previous studies [6]. Because the depths of demineralization had increased in groups A and B compared to the PCG, it can be assumed that the caries risk increases with the insertion of a multiband appliance using the bracket adhesive technique. It is interesting to note that deeper demineralization was observed in group B, whereas group A showed no significant difference compared to PCG. Comparing bracket-relevant groups A and B directly, the mean demineralization in group B was 7.29 μm less than in group A. Thus, in percentage terms, demineralization in group B was 9.59% less than in group A. Almosa et al. found, in this regard, that there could be a difference between various adhesive materials in the development of demineralization during bracket treatment [24]. Therefore, the suspicion is obvious that the insertion of a multiband appliance also increases the occurrence of demineralization in relation to the penetration depth and severity of demineralization. Because, in the present study, demineralization-free sections could only be microscoped in the NCG, the application of brackets with different bracket adhesive systems does not seem to have an effect on the number of demineralization.

Furthermore, a significance of *p* = 0.001 was shown in the comparison of severity grades in coronal and cervical lesions. Here, cervical lesions had a 2.65-fold higher probability of grade 2 demineralization compared to coronal lesions. For grade 3, the probability increased by a factor of 10.02. The cause is not evident from the available data, but it could be an increased tendency to erosion and abrasion of the cervical enamel. The thinner enamel running out here could lead to increased enamel cracks. The worse cervical hygienity compared to the coronal cusp discharge and, consequently, the more premineralized areas in the cervical enamel could favor the formation of more severe demineralization.

A limitation of the study is the in vitro experiment with the monoculture consisting of *S. sobrinus*, which represents a significantly simplified simulation of the otherwise very complex microbiological flora of the oral cavity. In the caries model used, *S. sobrinus* was chosen because it is a guide germ for surface adhesion in the oral cavity. Along with *S. mutans* and *Lactobacilli*, it is one of the leading germs in cariogenesis, but it has the highest virulence factor [25]. Furthermore, autoclaving the test specimens at 121 °C for 20 min does not correspond to the real clinical situation and the high temperature can have an influence on the enamel surface.

In addition, the hygienity of the bracket adhesives used in this trial represents a component with a major influence on demineralization in the bracket environment. Therefore, strictly speaking, the actual effects can only be determined by human clinical studies. Further studies should, therefore, be conducted to test and further develop materials for bacteria adhesion and subsequent demineralization of enamel in the bracket environment.

## 5. Conclusions

Both groups of brackets showed demineralization, which was more pronounced in comparison to non-adhered teeth. These results emphasize the importance of sufficient oral hygiene during multiband therapy. However, it was found that the nature of the dental material (the bracket adhesive) with which the bracket is applied to the enamel has an influence on bacterial accumulation and the resulting demineralization of the tooth structure. The test specimens of group B with conventionally bonded bracket adhesive showed significantly less (+10.8 μm) demineralization with regard to the penetration depth of the demineralization than the PCG. The difference in this respect to group A with the new bracket adhesive of FF brackets (+7.29 μm) was verifiable.

For final clarification of the full caries-protective potential of the modern FF bracket adhesive system, a series of tests under clinical conditions with implementation of a rinsing protocol should be undertaken, as the adhesive excess formed as a chamfer enables significantly improved hygienity. It seems plausible that new orthodontic bracket adhesive and the modern FF adhesive system could produce further positive results in such a study.

## Data Availability

Not applicable.
